# Why Are We by All Creatures Waited on?

**DOI:** 10.3201/eid1812.AC1812

**Published:** 2012-12

**Authors:** Polyxeni Potter

**Affiliations:** Author affiliation: Centers for Disease Control and Prevention, Atlanta, Georgia, USA

**Keywords:** art science connection, emerging infectious disease, art and medicine, Why Are We by All Creatures Waited on?, Lucas Cranach the Elder, Cardinal Albrecht of Brandenburg as St. Jerome, zoonoses, human–animal interactions, viruses, influenza, about the cover

**Figure:**
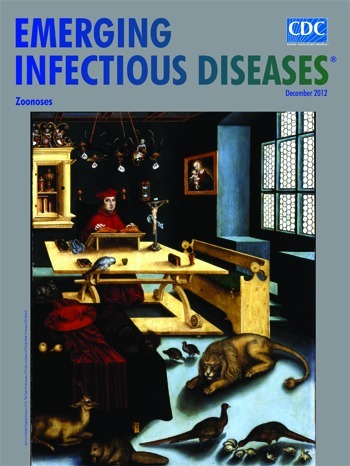
**Lucas Cranach the Elder (1472–1553) *Cardinal Albrecht of Brandenburg as St. Jerome* (1526) Oil on wood panel (114.9 cm × 78.9 cm)** John and Mable Ringling Museum of Art, The State Art Museum of Florida, a division of Florida State University, USA

Painter, engraver, designer of woodcuts, Lucas Cranach the Elder lived and worked during the last part of the Renaissance and embodied the integrative qualities valued by his age. In addition to accomplished artist, he was a successful entrepreneur, civic leader, and brilliant inventor. Among other innovations, he is credited with influencing the Danube school, a circle of painters along the Danube Valley, known for their advanced painterly style, printmaking, and etching; for early printing of woodcuts in color; for the full length portrait as an independent art form; and for various techniques intended to speed up painting and standardize technical processes.

Cranach’s early years and travels are sketchy. He was born in Germany’s Upper Franconia, the small town of Kronach, from which he took his name. He probably received the first art training from his father, the painter Hans Maler. A contemporary of two major artists from Germany, Matthias Grünewald and Albrecht Dürer, Cranach competed with them on local projects and patronage and often turned especially to Dürer’s work for inspiration. At age 30, he moved to Vienna, where he made influential associations with local humanists and painted two of his finest portraits. Some religious works showing appreciation of the beauty of nature, a characteristic of the Danube School, also date from this period. Soon he moved to Wittenberg to serve as court painter to Frederick the Wise, elector of Saxony, a position he retained for life under various electors.

Cranach became “one of the wealthiest burghers in Wittenberg.” He was elected city councilman several times and mayor twice, while fully engaged in painting, engraving, and directing all aesthetic needs of the court. He established and ran a prosperous studio that produced copies of his best works and all manner of decorative arts, from coin designs for the electorate to furniture. His sons, Hans and Lucas Cranach the Younger, both artists, worked in this studio and used his style so successfully that it is still difficult to fully authenticate what was done by his hand alone. The enterprise continued for decades after his death. His business acumen was such that he also ran a publishing press and had licenses to sell wine and own an apothecary.

Cranach’s greatest artistic contribution was his landscapes, which included elaborately detailed animals. He added to period art and Dürer’s naturalism an element of fantasy through the ornate treatment of forms. He also painted female nudes, whimsical mythologic scenes, and religious images containing contemporary everyday features. He is well remembered for his portraits, now a repository of the major figures of his age. He left behind perhaps the best portraits of Martin Luther. Other commemorated notables included Luther’s family and, despite Cranach’s own commitment to Protestantism, many Catholic clergy despised by Luther.

*Cardinal Albrecht of Brandenburg as St. Jerome*, on this month’s cover, is a culmination of many elements in Cranach’s work. This type of portrait, borrowing the image of a respected person to elevate that of another, was not unusual at this time. Dürer and others practiced it successfully, and many a churchman honored it. Invoking the virtues and protection of the saint in this portrait was Cardinal Albrecht, elector and archbishop of Mainz (1490–1545), a patron of artists and intellectuals as well as defender of the faith during the Counter-Reformation. Accused of extravagance and worldliness, he commissioned this portrait, in the guise of a religious icon, to proclaim his own beliefs and values. An admirer of St. Jerome, he wished to be likened to him. This revered saint, the most learned man of his age, was known not so much for his asceticism, which was without blemish, but for his knowledge―translating the Bible from Greek and Hebrew into Latin, a crowning literary achievement even by today’s standards.

At the time of St. Jerome (342–420), there were no cardinals in the Catholic Church. The iconography that would come to define the saint “… sitting in a chair, beside him that hat which cardinals wear nowadays and at his feet the tame lion,” was put forth by Giovanni d’Andrea, a canonical lawyer at the University of Bologna, in a biography of the saint and was perpetuated in later accounts. The lion legend behind the imagery goes all the way back to Aesop, but with the saint as the figure removing a thorn from the lion’s paw and forever gaining the beast’s loyalty and affection.

Jerome was a popular subject in art. Cranach alone painted at least eight images of the saint. The one on this month’s cover was patterned after Dürer’s effort on the same theme, a mirror image. Same small space, a cabinet or private room serving as a study or retreat—its mathematical perspective focusing on the main figure and the positioning of objects making the space appear larger; same ample window, allowing light and shadow effects; same antler-laden chandelier. Cranach and Dürer were colleagues, though Jerome is Cranach’s patron, the all-powerful cardinal. Somber, dour even, he sits ambiguously at the lectern, on a contemplative break from reading. His hat is shown in the foreground.

The painting is a treasure trove of symbolism, from the religious artifacts on the wall and in the cupboard behind the saint to the books, cups, fruit, hourglass, slippers, and other objects on and around the desk; and finally the animals, an unlikely menagerie, intended to symbolize characteristics laudable to the patron: monogamy, industriousness, frugality, loyalty, rejection of earthly desires. Thrown in with the other objects, the animals seem posed and indifferent to each other and their surroundings.

Despite the naturalism so valued in the art of Cranach’s time and his own interest in painting them, the animals in this portrait of St. Jerome remain just icons of human values. Stylized and scattered at the feet of the saint, they show none of their individuality or wildness. John Donne (1572–1631), a cleric as well as poet of the ages, aware of this two-dimensional treatment of animals, and not only in art, addressed what he saw as the inherent unfairness in perceiving them as strictly serving the interests of humans. In one of his holy sonnets, he asked outright, “Why are we by all creatures waited on?” and why do these creatures that are “more pure than I,” since they “have not sinned,” “provide food to me?”

Images of animals in art have changed, becoming more accurate and refined as humans were able to travel the world, see them in their natural habitat and study their anatomy and physiology. And the way animals are perceived by humans has changed science. Donne’s questions may not be entirely resolved but are eased by current understanding of animal–human phylogenetic closeness and knowledge of the zoonoses. Less an adversarial “them or us” issue, this current relationship is one of connectivity and sharing, on the physiologic as well as the emotional level. Many animals have gone from simply being domesticated to becoming members of human families and valued companions as pets. Others have come closer to humans as urbanization closed in on their habitat, and yet others have traveled far from their original nests. They all, too, on the zoonotic level, in the home and in the wild, share their infections freely and interact with humans outside traditional areas of exposure.

In this issue of the journal, human Hendra virus infection was acquired by close contact with horses infected by spillover from fruit bats, the natural reservoir for these viruses. MRSA organisms harboring a novel variant were detected in cats and dogs, which suggests that the variant is not restricted to human hosts. In Bangladesh, where HPAI H5N1 is endemic in poultry, live bird markets are a factor in human exposure. In the United States, agricultural fairs have been associated with bidirectional, influenza virus transmission between swine and humans. Fairgoers without routine occupational exposure to swine not only may be more susceptible to swine influenza viruses than those routinely exposed, they may also expose swine to a broader range of influenza A viruses for additional mixing. Identifying risk factors for transmission in both these venues would represent a step toward effective control.

Like Cranach’s animals, these human–animal interactions are symbolic of other, larger values, not moral and religious but biologic. Because, despite their surface unrelatedness, the interactions described in reports from around the world tie into one important common denominator, their zoonotic potential.
